# A randomized phase II/III study of adverse events between sequential (SEQ) versus simultaneous integrated boost (SIB) intensity modulated radiation therapy (IMRT) in nasopharyngeal carcinoma; preliminary result on acute adverse events

**DOI:** 10.1186/s13014-015-0472-y

**Published:** 2015-08-08

**Authors:** Anussara P. Songthong, Danita Kannarunimit, Chakkapong Chakkabat, Chawalit Lertbutsayanukul

**Affiliations:** Division of Radiation Oncology, Department of Radiology, Faculty of Medicine, Chulalongkorn University, King Chulalongkorn Memorial Hospital, 1873 Rama IV Rd, Pathumwan, Bangkok 10330 Thailand

**Keywords:** Intensity-Modulated Radiotherapy (IMRT), Simultaneous integrated boost, Nasopharyngeal carcinoma

## Abstract

**Background:**

To investigate acute and late toxicities comparing sequential (SEQ-IMRT) versus simultaneous integrated boost intensity modulated radiotherapy (SIB-IMRT) in nasopharyngeal carcinoma (NPC) patients.

**Methods:**

Newly diagnosed stage I-IVB NPC patients were randomized to receive SEQ-IMRT or SIB-IMRT, with or without chemotherapy. SEQ-IMRT consisted of two sequential radiation treatment plans: 2Gy x 25 fractions to low-risk planning target volume (PTV-LR) followed by 2Gy x 10 fractions to high-risk planning target volume (PTV-HR). In contrast, SIB-IMRT consisted of only one treatment plan: 2.12Gy and 1.7Gy x 33 fractions to PTV-HR and PTV-LR, respectively. Toxicities were evaluated according to CTCAE version 4.0.

**Results:**

Between October 2010 and November 2013, 122 eligible patients were randomized between SEQ-IMRT (54 patients) and SIB-IMRT (68 patients). With median follow-up time of 16.8 months, there was no significant difference in toxicities between the two IMRT techniques. During chemoradiation, the most common grade 3–5 acute toxicities were mucositis (15.4 % vs 13.6 %, SEQ vs SIB, *p = 0.788*) followed by dysphagia (9.6 % vs 9.1 %, *p = 1.000*) and xerostomia (9.6 % vs 7.6 %, *p = 0.748*). During the adjuvant chemotherapy period, 25.6 % and 32.7 % experienced grade 3 weight loss in SEQ-IMRT and SIB-IMRT (*p = 0.459)*. One-year overall survival (OS) and progression-free survival (PFS) were 95.8 % and 95.5 % in SEQ-IMRT and 98 % and 90.2 % in SIB-IMRT, respectively (*p = 0.472* for OS and *0.069* for PFS).

**Conclusion:**

This randomized, phase II/III trial comparing SIB-IMRT versus SEQ-IMRT in NPC showed no statistically significant difference between both IMRT techniques in terms of acute adverse events. Short-term tumor control and survival outcome were promising.

## Background

Nasopharyngeal carcinoma (NPC) is one of the most common head and neck tumors and has a good prognosis. The worldwide age-standardized incidence rates of NPC are 1.7 and 0.7 per 100,000 in males and females, respectively, whereas this increases to 6.4 and 2.4 in Southeast Asia. In Thailand, the age-standardized incidence rates of NPC are approximately 3.3 and 1.1 per 100,000 in males and females, respectively [1]. Radiotherapy is the standard treatment for NPC, due to its location and radiosensitivity.

In consideration of the proximity of many critical structures to the nasopharynx, including parotid glands, eyes, brain stem and spinal cord, it is challenging to provide an adequate dose to the tumor while sparing those surrounding tissues. An advanced radiotherapy technique, Intensity Modulated Radiation Therapy (IMRT), has been developed with the clear advantages of highly conformal and precise coverage with sharp dose gradients shown by many dosimetric studies; it has been impressively embraced in treatment of head and neck tumors [2–4]. In clinical studies, the use of IMRT can provide better preservation and faster recovery of parotid gland function [5–7].

IMRT may be applied in the same fractionation scheme as in conventional 3D Conformal Radiation Therapy (3D-CRT). Initially, the target volume and elective nodal regions are treated in the large field followed by a sequential boost to the gross tumor volume within the smaller field, the so called shrinking field technique or sequential technique (SEQ-IMRT). Subsequently, a new fractionation scheme has been initiated as an accelerated fractionation, known as simultaneous integrated boost IMRT (SIB-IMRT), which offers the opportunity to treat both primary and secondary targets simultaneously at different doses, so-called “dose painting” [8]. Compared to sequential treatment, SIB-IMRT uses only one radiation treatment plan for the entire course of treatment.

These two IMRT techniques are widely used in current practice [5–10]. According to dosimetric analysis, the target coverage from SEQ-IMRT and SIB-IMRT is the same, while SIB-IMRT provides superior dose distribution to the elective nodal area and better sparing of parotid glands and inner ear structures. Despite the promising results of SIB-IMRT for NPC, using a high dose per fraction to the target may result in higher dose per fraction to adjacent normal tissue, which can lead to more acute or late toxicity [11]. However, clinical studies comparing SIB-IMRT and sequential IMRT are scarce. Most clinical results of NPC treated by IMRT use the SIB technique [6, 11–18] and rarely compare with the sequential one.

The objectives of this study are to assess acute and late toxicities comparing SIB-IMRT with SEQ-IMRT and to evaluate clinical outcomes including tumor response, pattern of failure and survival of NPC patients treated with both IMRT techniques.

## Methods

### Patients and methods

Patients with newly diagnosed, histopathologic confirmed squamous cell carcinoma of the nasopharynx, stage I-IVB were eligible. Patients had to be at least 18 years of age with a Karnofsky performance status of at least 70 and adequate bone marrow, renal and liver function. The exclusion criteria included previous irradiation for a head and neck tumor or prior chemotherapy within six months, any other experimental therapeutic cancer treatment and any other malignancy except non-melanoma skin cancer or a carcinoma not of head and neck origin and controlled at least five years. Prophylactic use of amifostine or pilocarpine or concurrent use of amifostine, pilocarpine, diuretics, antidepressant or anticholinergic drugs that affect saliva secretion was prohibited.

All eligible patients received a pretreatment evaluation including complete history and physical examination, endoscopic biopsy, routine laboratory tests for hematologic, renal and hepatic function as well as a dental and nutritional evaluation prior to treatment. Radiological investigations consisted of computed tomography (CT) scan or magnetic resonance imaging (MRI) of the nasopharynx, chest radiography, ultrasound of the upper abdomen and bone scintigraphy. Positron emission tomography (PET) scan was optional. Pathologic confirmation of NPC was performed and re-classified according to WHO subtype [19].

### Study design

This study was a phase II/III randomized controlled trial approved by the institutional review board (IRB no.424/53) and patients provided written informed consent prior to the randomization. According to the effects of tumor and nodal burden of the disease on the adverse effects and clinical outcome [16], our patients were stratified by their T stages (T1-T3 versus T4) and N stages (N0-N1 versus N2-N3). The patients in each group were then randomized into two arms: the SEQ-IMRT arm and SIB-IMRT arm, using the stratified block randomization method.

### Treatment protocol

#### Radiotherapy

All patients were immobilized in the supine position with a tailored head-shoulder thermoplastic mask. A CT simulation was performed and transferred to the Eclipse planning system Version 8.6 (Varian Medical System, Palo Alto, CA). If available, MR images were co-registered with CT images. Target volume delineation was performed according to the RTOG 0225 protocol. Two planning target volumes (PTVs) were designed: PTV-High Risk (PTV-HR) and PTV-Low Risk (PTV-LR). The dose prescriptions were 70 Gy for PTV-HR at 2.12 Gy/fraction and 56 Gy for PTV-LR at 1.7 Gy/fraction delivered in 33 fractions for SIB-IMRT. For SEQ-IMRT, all PTVs were delivered at 2 Gy/fraction for 25 fractions with total dose of 50 Gy, followed by a boost of 20 Gy in 10 fractions to PTV-HR. The whole-field IMRT was applied in both techniques. Dosimetric difference, i.e., Biological Equivalent Dose (BED), between the two techniques is shown in Table 1.

**Table 1:** Dosimetric difference between SIB-IMRT and SEQ-IMRT strategies

Technique	BED (tumor)	BED (late)
SIB-IMRT (1-phase)		
PTV-HR = 2.12 x 33 = 70 Gy	84.8 Gy_10_	119.4 Gy_3_
PTV-LR = 1.7 x 33 = 56 Gy	65.6 Gy_10_	87.9 Gy_3_
SEQ-IMRT (2-phase)		
PTV-HR = (2 x 25) + (2 x 10) = 70 Gy	84.00 Gy_10_	116.7 Gy_3_
PTV-LR = 2 x 25 = 50 Gy	60.00 Gy_10_	83.3 Gy_3_

Biological effective dose (BED) calculation is based on the linear-quadratic model for cell kill with an α / β ratio of 10 for tumors and an α / β ratio of 3 for late-responding normal tissue

BED Total Dose (Gy) x (1 + dose per fraction(Gy))

(α / β)

Radiation treatment interruptions were allowed if there was any toxicity that affected patient’s safety or quality of life. If the treatment break exceeded 14 days, the patient was removed from protocol treatment but was then administered complete treatment at the discretion of the physician and was included in the study in the intention-to-treat analysis.

### Chemotherapy

Cisplatin, 40 mg/m^2^, was administered weekly concurrently with IMRT to those patients with more than T1 or positive nodal disease for the maximum of seven cycles. Subsequently, one month after completion of chemoradiation, adjuvant chemotherapy was given at four-week interval for three cycles. Each cycle consisted of cisplatin 80 mg/m^2^ and 5-FU 1000 mg/m^2^/ 24 h over a 96 h continuous infusion. In cases of toxicity, chemotherapy dosage modifications for the following cycles were based upon nadir counts and interim non-hematologic toxicities of the preceding cycle.

### Treatment monitoring and evaluation

Toxicity was reassessed at weekly intervals during chemoradiation, before each cycle of adjuvant chemotherapy and as regularly scheduled. Acute and late toxicities (defined as beyond three months after the completion of treatment) were recorded according to the Common Terminology Criteria for Adverse Events (CTCAE) version 4.03. CT or MRI of the nasopharynx was done at three months after the completion of chemoradiation to determine tumor response, using Response Evaluation Criteria in Solid Tumors (RECIST) criteria version 1.1. Fiberoptic nasopharyngeal examination was routinely performed 12 weeks after treatment completion.

### Statistical analysis

The primary end points of the treatment were acute and late toxicities. According to RTOG 0225 results in 57 NPC patients treated with SIB-IMRT with or without chemotherapy, severe acute adverse effects occurred in 78 % including 61.8 % grade 3, 11.8 % grade 4 and 4.4 % grade 5 [13]. Compared to our data, 73 NPC patients mainly (91.8 %) treated with SEQ-IMRT with carboplatin-based chemotherapy, severe acute toxicity (grade 3–5) occurred in 16.4 % of patients which was much lower than RTOG data [20]. To detect a 20 % difference between these two techniques with a power of 80 %, a p-value of 0.05 and 10 % drop out rate, a total of 218 patients were required. The investigators report here the preliminary results of 122 patients (56 %) accrued to date in this randomized phase III study. The secondary end points were to assess tumor response, pattern of failure and survival. Progression-free survival (PFS) was defined as the date of treatment initiation to the date of either disease progression or death. Overall survival (OS) was defined as the date of treatment initiation to the date of death, regardless of the cause.

Comparison of toxicity grading between treatment groups was statistically analyzed using Chi-square test. Survival analyses were computed using the Kaplan-Meier method and log-rank test. Statistical Packages for Social Sciences (SPSS) software version 17.0 was used for statistical analysis.

## Results

Between October 2010 and November 2013, a total of 130 patients of biopsy-proven NPC stage I-IVB was enrolled in this study. One hundred and twenty-two patients who completed chemoradiation and had followed up for more than 3 months were analyzed. Patient and disease characteristics are described in Table 2. Patients in SIB-IMRT had slightly more advanced disease; however, there was no statistically significant difference. Approximately 89 % of the patients had non-keratinizing undifferentiated squamous cell carcinoma. Median follow-up time was 16.8 months.

**Table 2:** Patient demographic and baseline characteristics (*N* = 122)

Characteristics	All (N = 122)	SEQ (N = 54)	SIB (N = 68)	*p*-value
Age, years				0.680
Mean ± SD	49.39 ± 10.30	49.7 ± 10.63	48.96 ± 9.96	
Sex				0.563
Male	95 (77.9 %)	39 (72.2 %)	56 (82.4 %)	
Female	27 (22.1 %)	15 (27.8 %)	12 (17.6 %)	
Performance status				-
90–100	122 (100 %)	54 (100 %)	68 (100 %)	
WHO classification				0.983
Type I (Keratinizing SCCA)	2 (1.6 %)	1 (1.9 %)	1 (1.5 %)	
Type IIA (NK, diff. SCCA)	11 (9.0 %)	5 (9.2 %)	6 (8.8 %)	
Type IIB (NK,undiff. SCCA)	109 (89.4 %)	48 (88.9 %)	61 (89.7 %)	
T stage				0.741
1	35 (28.7 %)	17 (31.5 %)	18 (26.5 %)	
2	50 (41.0 %)	22 (40.7 %)	28 (41.2 %)	
3	24 (19.7 %)	11 (20.4 %)	13 (19.1 %)	
4	13 (10.6 %)	4 (7.4 %)	9 (13.2 %)	
N stage				0.910
0	5 (4.1 %)	2 (3.7 %)	3 (4.4 %)	
1	22 (18.0 %)	9 (16.6 %)	13 (19.1 %)	
2	77 (63.2 %)	36 (66.7 %)	41 (60.3 %)	
3	18 (13.8 %)	7 (13.0 %)	11 (16.2 %)	
AJCC Stage grouping				0.259
II	18 (14.8 %)	8 (14.8 %)	10 (14.7 %)	
III	75 (61.4 %)	37 (68.5 %)	38 (55.9 %)	
IVA	11 (9.0 %)	2 (3.7 %)	9 (13.2 %)	
IVB	18 (14.8 %)	7 (13.0 %)	11 (16.2 %)	
Mean PTV volume, cc				0.432
PTV-HR ± SD	366.29 ± 122.07	354.76 ± 105.61	376.68 ± 135.70	
PTV-LR ± SD	813.7 ± 150.52	802.98 ± 146.84	823.37 ± 154.98	0.558
RT duration, days				0.002
Mean ± SD	49.36 ± 3.72	50.54 ± 3.87	48.43 ± 3.33	

*AJCC* American Joint Committee of Cancer, *SEQ* Sequential IMRT arm, *SIB* Simultaneous integrated boost IMRT arm, *PTV* Planning target volume, *HR* High risk region, *LR* low-risk region

Dosimetric data were summarized in Table 3. There was a statistically significant difference in average PTV-LR dose and maximal dose to spinal cord and eyes as well as median dose to one parotid gland and cochlear between the two treatment arms. The physical PTV-LR dose difference resulted from the different prescription doses between the two techniques with regards to BED as shown in Table 1.

**Table 3:** Dosimetric comparison of two IMRT techniques

Target / Organ at risk	Average dose (Mean ± SD, Gy)		*p*-value
	SEQ	SIB	
PTV-HR dose (D95%)	69.80 ± 2.66	69.47 ± 2.00	0.452
PTV-LR dose (D95%)	52.21 ± 3.22	56.71 **±** 2.07	0.000
Maximal spinal cord dose (D1cc)	40.65 ± 4.34	42.09 **±** 2.23	0.030
Maximal brain stem dose (D1cc)	48.84 ± 6.02	50.13 ± 4.44	0.193
Median dose of one parotid gland (D50%)^a^	24.44 ± 8.69	21.91 ± 4.04	0.035
Maximal optic nerve dose	30.06 ± 13.99	34.26 ± 16.58	0.132
Median cochlear dose (D50%)	47.26 ± 8.52	44.01 ± 7.84	0.032
Maximal eye dose	19.40 ± 7.34	23.05 ± 9.10	0.016
Maximal lens dose	6.44 ± 1.78	7.91 ± 1.07	0.149
Maximal mandible dose	71.27 ± 5.22	71.02 ± 5.74	0.802
Median oral cavity dose (D50%)	37.28 ± 4.21	36.40 ± 4.30	0.256
Median vocal cord dose (D50%)	39.55 ± 6.16	40.97 ± 4.85	0.667

^a^The smallest dose between bilateral parotid glands

### Treatment compliance

**Fig. 1:**
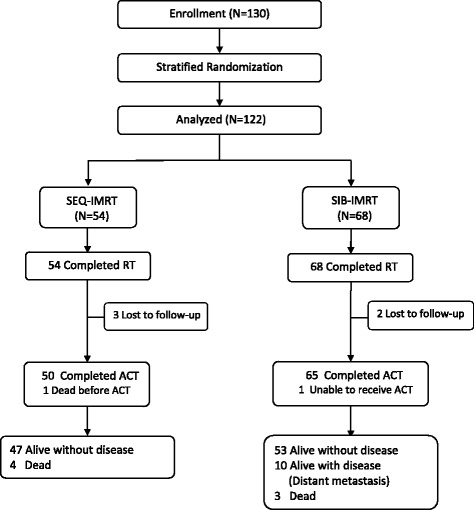
CONSORT diagram showing the flow of participants. Abbreviation: RT = Radiotherapy, ACT = Adjuvant Chemotherapy

The CONSORT diagram of treatment is presented in Fig. 1. Treatment interruption occurred in two patients from each arm due to grade 3 neutropenia and a flooding disaster in Thailand; the total radiation durations of these patients were 61 and 63 days, respectively. Of the 122 patients, 25 patients (20.5 %) completed seven cycles of concurrent weekly cisplatin and 115 patients (94.3 %) received at least four cycles; 52 patients (96.3 %) and 63 patients (92.6 %) in SEQ-IMRT and SIB-IMRT, respectively. One patient in SEQ-IMRT received radiation alone due to advanced age and died before chemotherapy. Eighty patients (65.6 %) completed all three cycles of adjuvant chemotherapy; 32 patients (59.3 %) and 48 patients (70.6 %) in SEQ-IMRT and SIB-IMRT, respectively. The reasons contributing to discontinuation during treatment included treatment-related toxicities; for example, prolonged grade 2 neutropenia, severe nausea and anorexia. One patient in SIB-IMRT was unable to receive adjuvant chemotherapy due to prolonged neutropenia.

### Acute and late toxicity

We classified toxicities into acute and late toxicities by three-month periods after completion of the adjuvant chemotherapy. All patients experienced some degrees of acute toxicities, most of which were mild severity (grade 1–2). Severe toxicities (grade 3–5) during concurrent chemoradiation occurred in 18 patients (14.7 %), 9 patients (16.7 %) in SEQ-IMRT and 9 patients (13.2 %) in SIB-IMRT (*p-value = 0.788*), most of which was oral mucositis. One treatment-related death was suspected in a 79 year-old man in SEQ-IMRT due to severe oral mucositis and electrolyte imbalance.

During the adjuvant chemotherapy period, the incidence of acute grade 3 weight loss (more than 20 % from baseline) was 10 patients (20 %) in SEQ-IMRT and 18 patients (27.3 %) in SIB-IMRT (*p-value = 0.459*). However, there was no significant difference in severity of acute toxicity during both treatment periods between the two techniques, as described in Table 4.

**Table 4:** Severe (grade 3–5) acute toxicity during concurrent chemoradiation and adjuvant chemotherapy

	SEQ	SIB	*p*-value
Severe acute toxicity during concurrent chemoradiation			
Weight loss	5.9 %	6.2 %	1.000^*^
Oral mucositis	15.4 %	13.6 %	0.788
Dysphagia	9.6 %	9.1 %	1.000^*^
Xerostomia	9.6 %	7.6 %	0.748^*^
Dermatitis	7.7 %	9.1 %	1.000^*^
Severe acute toxicity during adjuvant chemotherapy			
Weight loss	20 %	27.3 %	0.459
Dysphagia	2 %	1.5 %	1.000^*^

^*^Fischer’s exact test was used due to low expected count data

During follow-up after the completion of adjuvant treatment, the incidence of grade 3 weight loss was 8 patients (26.7 %) in SEQ-IMRT and 15 patients (34.9 %) in SIB-IMRT, *p-value* = 0.457. None of patients experienced grade 3–4 gastrointestinal, dermatologic and hematologic toxicities during follow-up. Neither renal function impairment nor treatment-related death was found. In the three-month period, rates of grade 1 and grade 2 xerostomia were 24.3 % and 9.5 %, respectively. No patient experienced more than grade 2 xerostomia. Overall, there was no significant difference in severity of late toxicity between SEQ-IMRT and SIB-IMRT.

### Tumor response

Of the 122 patients, 115 patients could be evaluated for response by performing a CT scan of the nasopharynx at three months after completion of radiation. Five patients were lost to follow up and 2 patients died before evaluation. According to RECIST criteria version 1.1, 41 patients (83.7 %) in SEQ-IMRT and 56 patients (84.8 %) in SIB-IMRT achieved complete remission. Eight patients (16.3 %) and 10 patients (15.2 %) had partial response in SEQ-IMRT and SIB-IMRT, respectively. One patient had residual primary disease and received additional boost with photon of 3Gy/fraction for 10 fractions at 5 months after complete chemoradiation. Seventeen patients had residual cervical lymph nodes and received additional boost with 6–9 MeV electron 3 Gy/fraction for 5 fractions. Three of them further received salvage neck dissection but all were pathologically negative.

### Pattern of failure

Locoregional recurrence was found in only one patient (1.5 %) in SIB-IMRT. None in SEQ-IMRT developed distant metastasis while this occurred in nine patients (13.6 %) in SIB-IMRT *(p-value = 0.005)*. Seven patients (5.7 %) died.

### Survival outcome

**Fig. 2:**
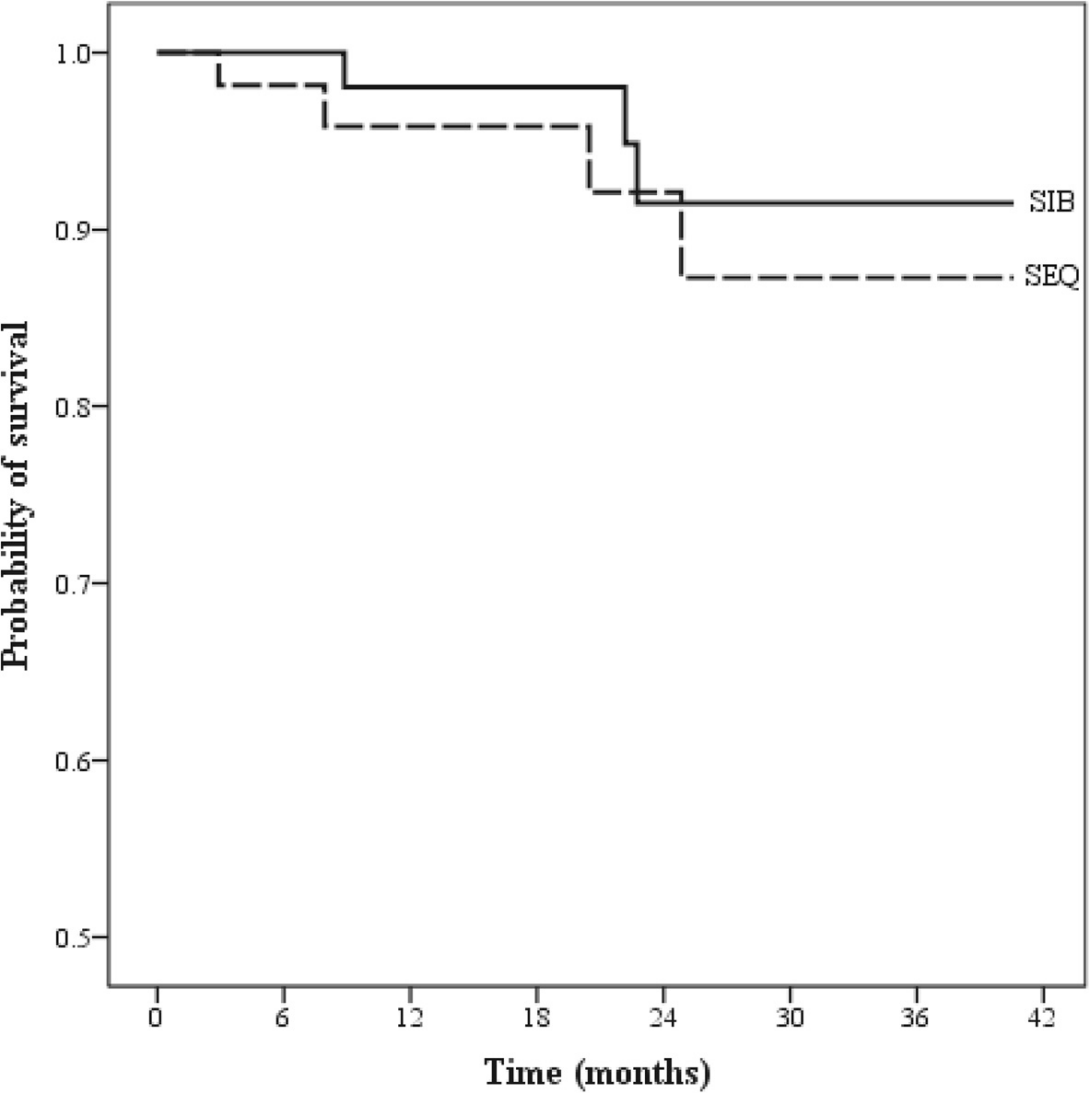
Overall survival between two IMRT techniques

**Fig. 3:**
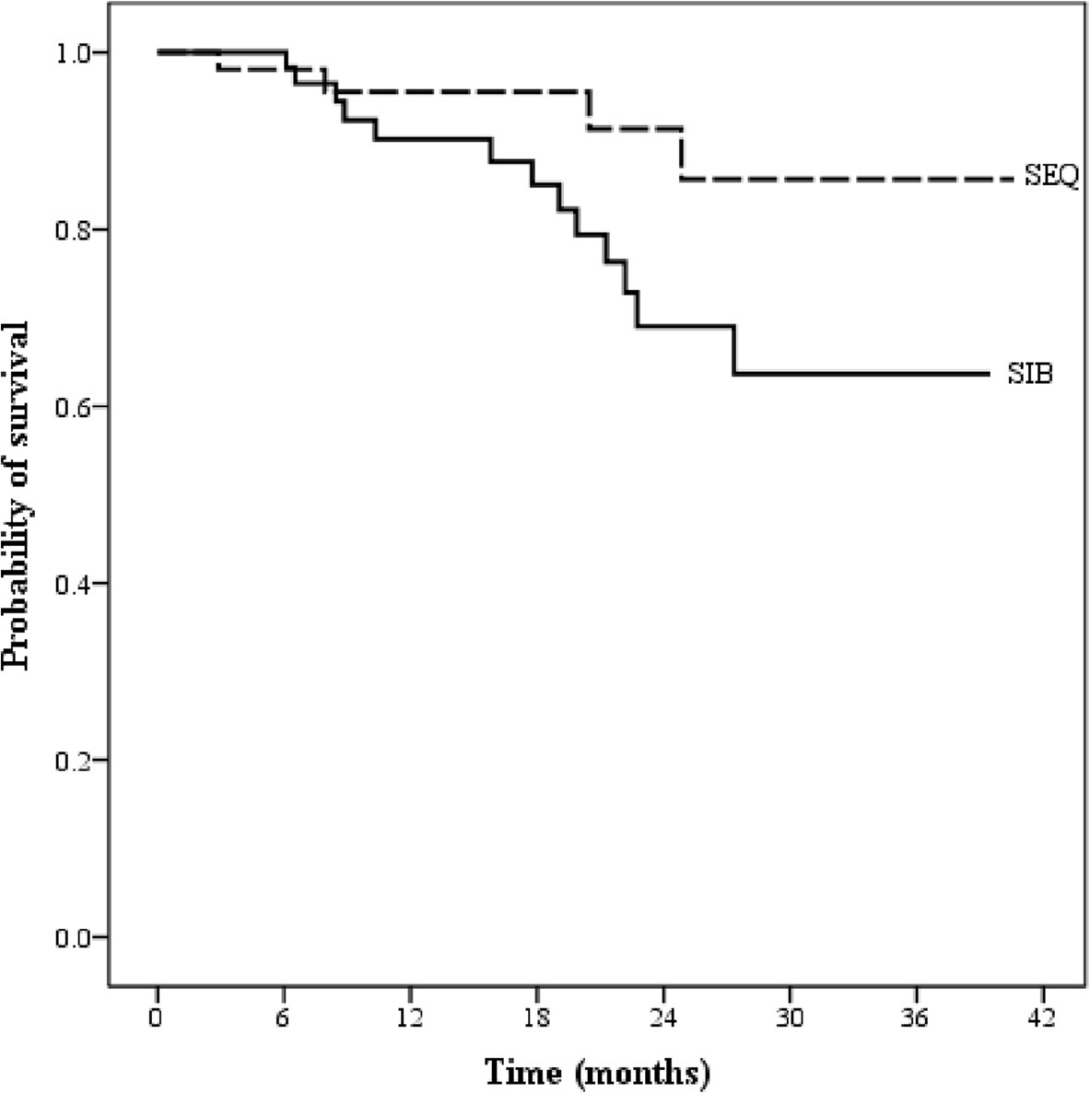
Progression-free survival between two IMRT techniques

Survival analysis was performed using Kaplan-Meier survival analysis, as shown in Figs 2 and 3. For overall and in both treatment groups, the median survival was not reached at the time of analysis. One-year overall survival (OS) and progression-free survival (PFS) were 95.8 % and 95.5 % in SEQ-IMRT and 98 % and 90.2 % in SIB-IMRT (*p-value = 0.472 for OS and 0.069 for PFS*), respectively.

## Discussion

Radiotherapy with or without chemotherapy has been the standard treatment for nasopharyngeal carcinoma [21]. Several studies have shown the popularity and benefits of the advanced radiation technique, IMRT, which can achieve dose escalation to the tumor and also reduce the dose to the surrounding normal tissue [2–4]. IMRT may be applied in either sequential (SEQ) technique or simultaneous integrated boost (SIB) technique. SIB-IMRT needs only a single treatment plan, which is more convenient and more efficient than SEQ-IMRT. From a socioeconomic prospective, fewer treatment fractions (i.e., 33 fractions in SIB-IMRT as compared to 35 fractions in SEQ-IMRT) also lead to time and cost savings as well as reducing the workload of heath care providers. Moreover, from the radiobiological point of view, reduction in overall treatment time is assumed to reduce the risk of tumor clonogens regrowth during the late phase of radiation treatment and probably to improve tumor control probability [22]. In our study, the BED to PTV-HR (PTV-70) was slightly higher in SIB-IMRT than SEQ-IMRT, 84.8 Gy_10_ and 84.0 Gy_10_, respectively. This higher fraction size might have an effect on cellular death and regeneration of acutely responding normal tissue surrounding target volumes. Denham reported an earlier time of adverse effects during accelerated RT than conventional RT [23].

Chen [11] and Nesrin [24] performed dosimetric studies comparing the target volume coverage and normal tissue sparing of SIB-IMRT versus SEQ-IMRT for nasopharyngeal carcinoma. Both of them found that target coverage was the same for both techniques while SIB-IMRT provided superior dose distribution to the elective nodal area and better sparing of the parotid glands and inner ear structures. This is the same direction in our study which showed that the median dose to one parotid gland and cochlear in SEQ-IMRT (24.44 ± 8.69 Gy and 47.26 ± 8.52 Gy) were higher than in SIB-IMRT (21.91 ± 4.04 Gy and 44.01 ± 7.84 Gy) *(p = 0.035* for parotid and *p = 0.032* for cochlear*)*. Additionally, the median oral cavity dose was higher in SEQ-IMRT despite no significant difference (*p = 0.256*). The possible explanation was the sum of two treatment plans in SEQ-IMRT resulted in expansion of low-dose area. However, it was also noted that critical organs close to the gross target volume, brain stem and spinal cord as well as optic nerves and eyes especially in T4 cases, might receive higher doses when applying SIB-IMRT due to higher prescription dose to PTV-LR in SIB-IMRT. However, the dose to these organs was within acceptable constraint. We found that the small difference of parotid dose between SIB and SEQ-IMRT did not translate to different clinical outcome.

Most clinical studies and a review of SIB-IMRT in NPC demonstrated excellent clinical outcomes in terms of tumor control and acceptable acute and late toxicities [12–18, 25]. However, clinical studies comparing SIB-IMRT and SEQ-IMRT are scarce. In our institute, SEQ-IMRT and SIB-IMRT have been used in the treatment of nasopharyngeal cancer since 2005, according to physicians’ preference. Lertbutsayanukul initially used SEQ-IMRT and reported an 89 % complete response rate in 18 head-and-neck cancer patients [10]. In this study, 83.7 % and 84.8 % of the patients in SEQ-IMRT and SIB-IMRT had complete remission.

We used concurrent weekly cisplatin since it has been proven for its efficacy, better compliance and convenience as compared to three-weekly regimens [26]. For adjuvant chemotherapy, cisplatin 80 mg/m^2^ on Day 1 combined with 5FU 1000 mg/m^2^/day for five days was delivered monthly to a total of three cycles as the intergroup 0099 protocol [21]. Among 122 patients in our study, 115 patients (94.3 %) received at least four cycles of concurrent chemotherapy, compared with 78 % in the study of Chan [26], which was higher than a three-week regimen in other studies. Most of our patients (65.6 %) completed three cycles of adjuvant chemotherapy, which was higher than the compliance rate in intergroup 0099 [21] and RTOG 0225 [13] studies, at 55 % and 46.5 %, respectively.

Our primary outcome was to assess acute and late toxicities using Common Terminology Criteria for Adverse Events (CTCAE) version 4.03. All patients experienced some degrees of acute toxicities, most of which were of mild severity, grade 1–2. Weight loss and oral mucositis were the main problems during treatment. No significant difference in acute and late toxicities between two IMRT techniques was observed.

Because different criteria were used for the toxicity evaluation, comparing the toxicity among several published trials might not be appropriate. In RTOG 0225 which used similar toxicity evaluation criteria, acute grade 3 mucositis, defined as confluent pseudomembranous reaction (contiguous patches generally >1.5 cm in diameter) in CTCAE version 2.0 and as severe pain or interfere oral intake in CTCAE version 4.03, was 29.4 % in the RTOG study [13] compared to 13.9 % in our study. There was one patient who experienced acute grade 5 mucositis in both trials, accounting for 4.4 % and 0.8 % in RTOG study and our study, respectively. Grade 3 or more acute mucositis in our study is less than in the others, thanks to the dose volume constraint we put to the oral cavity.

The rate of grade 3 dermatitis, which was defined as confluent moist desquamation ≥1.5 cm diameter and not confined to skin folds or pitting edema in CTCAE version 2.0 and as moist desquamation other than skin folds and creases or bleeding induced by minor trauma or abrasion in CTCAE 4.03, was 13.2 % in the RTOG study [13] compared to 8.2 % in our study.

Late grade 2 xerostomia in the three-month period in our study was 9.5 % compared to 64 % reported by UCSF [5]. At 12 months, late grade 2 xerostomia in our study was only 0.8 % compared with 32 % and 13.5 % in MSKCC [6] and RTOG 0225 [13] studies, respectively. Of note were the different toxicity criteria among these studies.

Regarding significant anatomic changes during RT, including tumor shrinkage and changes in body contour due to weight loss, these factors can affect the dose distribution to the target volume and normal tissue, which can result in inadequate target coverage or increased toxicities. Notably, the proportion of T4 and N3 staging, PTV volume as well as the incidence of weight loss during CCRT was slightly higher in SIB-IMRT thus an increased toxicity in SIB-IMRT was possible. However, the current preliminary data did not show the significant difference between both IMRT techniques. Several studies reported the excellent outcomes on disease control and late toxicity of adaptive radiation treatment in NPC patients, especially those with advanced T and N stage [27]. Therefore, we anticipate that patients with larger PTV volume treated with SEQ-IMRT would get better results from two treatment-planning with re-CT simulation after half way of treatment.

With a median follow-up time of 16.8 months, locoregional recurrence was found in only one patient and distant metastasis occurred in nine patients in SIB-IMRT. On analysis of tumor response, pattern of failure and survival, there was no significant difference between the two different IMRT techniques, except for the rate of distant metastasis *(p = 0.005)*. The higher distant metastasis rate in SIB-IMRT might be explained by higher proportion of T4, N3 and stage IV disease despite insignificant difference between both treatment arms.

The limitation of this study was that it was non-blinded, as the same physicians were responsible for each subject through the process of treatment planning and follow-up, thus they were not blinded from treatment group, resulting in possible observer bias. Neither was the patients blinded because of different numbers of radiotherapy fractions. Additionally, the diagnostic techniques for staging and re-staging used in this trial was mainly CT scan of the nasopharynx and chest xray instead of MRI of the nasopharynx and CT scan of the chest, respectively, due to technical availability limitations. However, patients whom were of potential benefit from these techniques, such as T4 patients and those with abnormal x-ray, would be strongly considered. Furthermore, as this is a preliminary result we plan to allocate and randomize additional patients in order to reach significant statistical analysis. Long-term follow-up is needed to demonstrate efficacy on disease control and survival as well as late toxicities.

## Conclusion

Preliminary results from this study showed that SEQ-IMRT has comparable toxicity to SIB-IMRT. Short-term tumor control was promising. However, more patient accrual and longer follow-up are needed.

## Abbreviations

3D-CRT: 3D conformal radiation therapy
BED: Biological equivalent dose
CT: Computed tomography
CTCAE: Common terminology criteria for adverse events
MRI: Magnetic resonance imaging
NPC: Nasopharyngeal carcinoma
OS: Overall survival
PET: Positron emission tomography
PTV-LR: Low-risk planning target volume
PTV-HR: High-risk planning target volume
PFS: Progression-free survival
RECIST: Response evaluation criteria in solid tumors
SEQ-IMRT: Sequential intensity modulated radiation therapy
SIB-IMRT: Simultaneous integrated boost intensity modulated radiation therapy
SPSS: Statistical Packages for Social Sciences
WHO: World Health Organisation
